# Effect of rhBMP-2 applied with a 3D-printed titanium implant on new bone formation in rabbit calvarium

**DOI:** 10.1590/1678-7757-2020-1092

**Published:** 2021-09-08

**Authors:** RYU Jaeyoung, Hyo-Sun KANG, Byung-Hun KANG, Seunggon JUNG, Min-Suk KOOK, OH Hee-Kyun, Ji-Yeon JUNG, Hong-Ju PARK

**Affiliations:** 1 Chonnam National University Department of Oral and Maxillofacial Surgery Gwangju Republic of Korea Chonnam National University, Department of Oral and Maxillofacial Surgery, Gwangju, Republic of Korea.; 2 Chonnam National University Department of Oral Physiology Gwangju Republic of Korea Chonnam National University, Department of Oral Physiology, Gwangju, Republic of Korea.

**Keywords:** Three-dimensional printing, Bone morphogenetic protein, Osteogenesis

## Abstract

**Objective:**

This study sought to compare the biocompatibility of a three-dimensional (3D)-printed titanium implant with a conventional machined titanium product, as well as the effect of such implant applied with recombinant human Bone Morphogenetic Protein Type 2 (rhBMP-2) for guided bone regeneration.

**Methodology:**

Disk-shaped titanium specimens fabricated either by the conventional machining technique or by the 3D-printing technique were compared by MC3T3-E1 cells cytotoxicity assay. New bone formation was evaluated using a rapid prototype titanium cap applied to the calvaria of 10 rabbits, which were divided into two groups: one including an atelopeptide collagen plug on one side of the cap (group I) and the other including a plug with rhBMP-2 on the other side (group II). At six and 12 weeks after euthanasia, rabbits calvaria underwent morphometric analysis through radiological and histological examination.

**Results:**

Through the cytotoxicity assay, we identified a significantly higher number of MC3T3-E1 cells in the 3D-printed specimen when compared to the machined specimen after 48 hours of culture. Moreover, morphometric analysis indicated significantly greater bone formation at week 12 on the side where rhBMP-2 was applied when evaluating the upper portion immediately below the cap.

**Conclusion:**

The results suggest that 3D-printed titanium implant applied with rhBMP-2 enables new bone formation.

## Introduction

Maxillofacial bone defects may account for several reasons, including trauma, malformation, tumors, or infectious diseases. Even when the original condition is solved, reconstructing the defect and recovering function in these cases has always been a challenge.^1^ Although the literature agrees that bone grafts is the gold standard for the reconstruction of bone defects, the additional procedure to harvesting autologous bone incur several limitations. Thus, many studies have actively investigated materials or tissue engineering capable of inducing bone regeneration, including bone substitutes such as allogeneic, xenogeneic, and synthetic bone. Bone morphogenetic protein (BMP) is yet another factor widely known by its potential for promoting bone regeneration.^2^

Being the representative technology of the fourth industrial revolution, three-dimensional (3D) printing is characterized by its custom design output.^3^ Printouts can be obtained through various materials and different types of printing – such as bioprinting, which combines 3D biomaterial scaffolds, cells, and signaling molecules to regenerate tissue.^4^Bone-regeneration scaffold requires biocompatibility, mechanical properties, and structure for cell survivability.^5^and the most common titanium materials in recent years are

Titanium alloy Ti6Al4V (Ti-64) and commercially pure titanium (CP–Ti) are the most common titanium materials used in guided bone regeneration in recent years.^6^Depending on the laser power or layer thickness, each 3D printing method has different properties.^7^Considering the aforementioned titanium materials, the most widely known method for 3D printing is the powder bed fusion (PBF), such as selective laser sintering (SLS), direct metal laser sintering (DMLS), selective laser melting (SLM), and electron beam melting (EBM) – among which the DMLS method with titanium and titanium alloy has been a useful manufacturing technique.^8^This is due to its reproducibility, customization, controllable surface geometry, cost-effectiveness, and relatively good resolution (~20mm), sparing interest on its application to implantable and prosthetic devices.^9^

Many studies have recently investigated 3D-printed titanium implants. However, studies addressing updated the biocompatibility, appropriate design, and accuracy of titanium metal printouts are still scarce, as well as those on its structural stability and bone regeneration efficiency after implantation *in vivo* – thus indicating a fertile ground for research. Although recent studies related to 3D-printed titanium implants focused on developing a lattice or porous scaffold for bone regeneration, more complex structures demand a more elaborate output evaluation, just as finer units demand a more continuous stability evaluation.^10^

The literature shows that BMP plays an important role in bone regeneration, being recently employed in diverse bioprinting research.^4^However, these researches aimed to apply bone-inducing materials to the printouts of complex structures used as scaffolds, which requires a large set of variables in the analyses. Thus, this study compares the biocompatibility and structural stability of a 3D-printed titanium implant with a machined titanium structure when used as an outer skeletal structure. We also sought to determine the effect of a 3D-printed cap added with a bone-inducing material and BMP on bone regeneration.

## Methodology

### Experimental material

**Figure 1:**
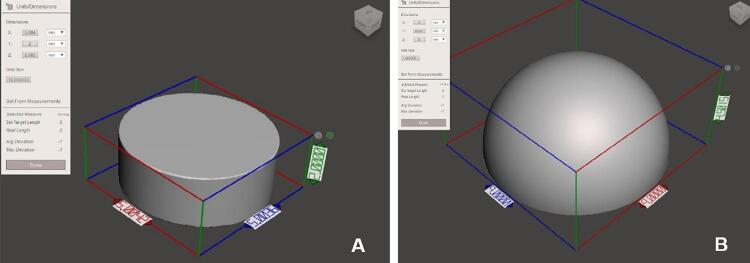
Computer-assisted design of the titanium disk

The disks and caps used in the animal experiment were designed with the Autodesk Meshmixer software (San Rafael, CA, USA): the 3D-printed titanium implant in a 2-mm thick disk shape of 5mm diameter and the cap-shaped rapid prototype (RP) in a hemispherical shape according to the size of the trephine bur, with 9mm of diameter, 4.5mm of height, and 0.8mm of thickness (Figure 1). The materials were then fabricated through a direct metal printing (DMP) technique, using Ti-6Al-4V powder and a ProX 100 dental 3D printer (3D Systems, Rock Hill, SC, USA).

(A, diameter, 5mm; height, 2mm) and cap (B: diameter, 9mm; height, 4.5mm; thickness, 0.8mm) for 3D printing.

The ready-made titanium material consisted of an off-the-shelf titanium plate of the Universal Orthognathic Internal Fixation System (Stryker Craniomaxillofacial, Kalamazoo, MI, USA) cut into a 2-mm thick disk shape of 5mm diameter. Each specimen was ultrasonically cleaned three times, dried, and sterilized with ethylene oxide (EO) gas.

### Experimental method

#### Cytotoxicity assay

In total, 14 specimens were prepared – seven 3D-printed and seven ready-made titanium products. Their biocompatibility was compared using a cytotoxicity test that evaluated the cell response of each group through MC3T3-E1 cells (calvaria newborn mouse derived, ATCC CRL2593), which were cultured in minimum essential medium alpha modification (α-MEM, Gibco, Grand Island, NY, USA) supplemented with 10% fetal bovine serum and 1% solution of 100 U/ml penicillin and 100 mg/ml streptomycin (Lonza, Basel, Switzerland). Cell viability was measured using the Enhanced Cell Viability Assay Kit (EZ-Cytox, DoGenBio, Seoul, Korea) according to the manufacturer’s protocol. The sterilized samples were placed in a 20-well cell culture plate, received the MC3T3-E1 cells (1x103cells), and were incubated. After 24 hours the medium was replaced with new medium containing water soluble tetrazolium salt (WST) solution, and after 48h the absorbance was measured in a microplate reader at 450nm with a 630nm reference wavelength.

## Animal experiment

Experiments involving animals were performed in accordance with all applicable international, national, and institutional guidelines for the care and use of animals. The project was approved by the Regional Animal Ethics Committee (CNU IACUC-H-2018-30).

The study sample consisted of ten mature male rabbits weighing 2.0 to 2.5kg, whose skulls were divided into the left and right sides of the sagittal suture line. The health/immune status, genetic modification, and genotype of all animals were verified, as well as the history of any previous procedures, in which case the animal was excluded. Animals underwent general anesthesia using a 200-mg ketamine hydrochloride (Ketalar™, Pfizer, Surrey, UK) injection applied to both gluteus maximus muscles. All rabbits had their head hair shaved and their skin disinfected with chlorhexidine to secure the field of vision and ensure an aseptic environment. Then, 2% lidocaine containing 1:100,000 epinephrine was topically injected into the surgical area.

To position the RP titanium cap into the calvaria bone, an incision was made along the sagittal suture line of the skull using a no. 15 blade, elevating and reflecting the periosteum. Using a trephine bur (outer diameter 9mm), a circular groove was formed on both sides of the sagittal suture at a 1-mm depth to prevent cap displacement and increase its stability. To induce calvaria bone marrow bleeding, a perforation was made on the center of the groove using a bur.

Animals were then divided into two groups: in group I (n=10), the RP titanium cap received a collagen plug (Ateloplug^®^, Bioland, Cheongwon, Korea); whereas in group II (n=10) it received a collagen plug soaked with lyophilized E. coli-derived rhBMP-2 (Cowellmedi Co., Busan, Korea) mixed with distilled water at a concentration of 0.1 mg/ml. After copious irrigation of the surgical site, the periosteum was sutured with 4-0 Vicryl^®^ and the skin with ٦-٠ Ethylon^®^, being sterilized with potadine. Once awake, animals were transferred to a single breeding room to avoid confounders. Cases in which animals died before the end of the experiment or the specimen was not properly collected for analysis were excluded from the study.

**Figure 2:**
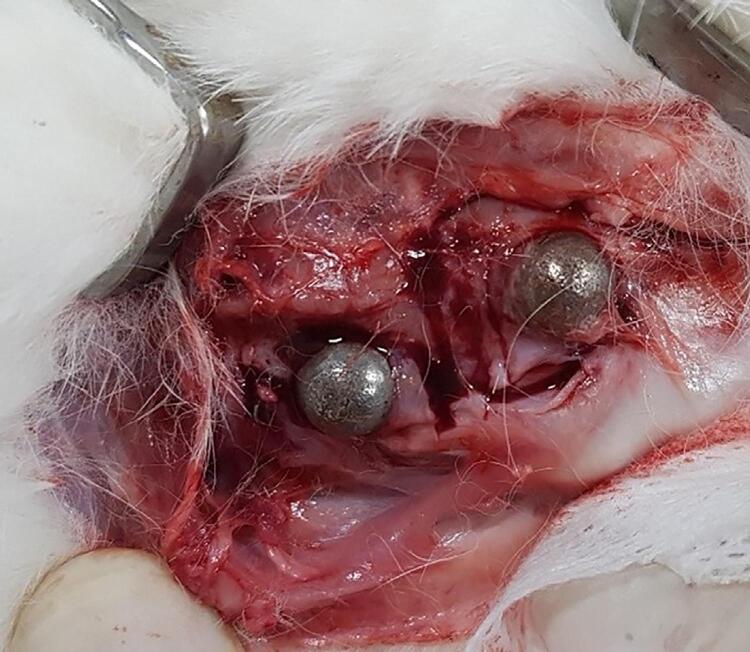
Photograph at harvesting specimen during the animal experiment. The cap positions were well maintained

Animals were euthanized at either six or twelve weeks after the procedure, and the implant and adjacent conditions were inspected (Figure 2). Specimens were separated from the 3D-printed titanium cap and subjected to radiological and histological analysis. Different researchers were responsible for conducting animal experiments and specimen analysis, and those responsible for the analysis were blinded to experimental groups to avoid bias.

## Radiographic inspection with micro-computed tomography (CT) and volumetric analysis

Specimens underwent radiographic inspection using a Quantum GX micro-CT imaging system (Perkin Elmer, Hopkinton, MA, USA) with 90kV source voltage, 80mA source current, 50mm voxel size, 0.5° rotation angle, and 4-minute scan time. The volume of new bone formed was measured using the In vivo5 Anatomage (San Jose, CA) software and expressed in Hounsfield units (HU) – the whole volume under the cap first, followed by new bone formed within 2mm beneath the cap. The analyses were performed on HU scales of more than 300 and 1000, respectively. Volume measurements were repeated twice with an interval of at least one week, and the intraclass correlation coefficients (ICC) were also investigated. Statistical differences in the volume between the groups were analyzed using the Mann-Whitney U test. Statistical analysis was performed using the Statistical Package for the Social Sciences (SPSS) software Version 26^®^ (IBM Corp., Armonk, NY, USA), and p-values <0.05 were considered statistically significant.

## Histological analysis

After micro-CT scans, tissue specimens were fixed in 10% formalin solution for two days and paraffin-embedded by traditional methods. Then, 5-µm sections were prepared and dyed with hematoxylin and eosin. Histological examination was performed via optical microscopy (Nikon, Melville, NY, USA) with the Aperio ImageScope software version 9.1 (ImageScope, Aperio Technologies, Vist, CA, USA), providing histological digital images.

## Results

### Cytotoxicity assay

**Figure 3:**
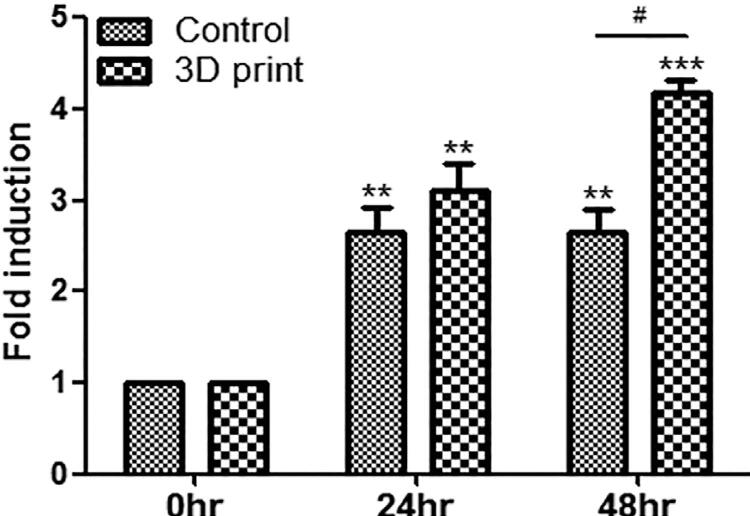
Results from cell viability tests (n=3)

The RP titanium disk showed a higher number of cells in the medium cultured for 24 hours when compared to the ready-made titanium plate, but without significant difference. In the ready-made plate, cell count at 48 hours was similar to that observed in the 24-hour culture. On the other hand, the RP disk showed a larger number of cells, significantly different than those in the ready-made plate (Figure 3).

MC3T3-E1 cell counts between the titanium plate (control) and the RP titanium disk (3D print) specimen were significantly different after 48 hours (p< 0.05). Statistical comparisons were performed by the Student's t-test (#, p<0.05; **, p<0.01; ***, p<0.001).

### Animal experiment

One rabbit died during the experiment, so that four animals were euthanized at six weeks postoperatively and five at 12 weeks. By observing changes in the shape and surrounding bones, we verified that most of the specimens were well-fixed on the calvaria and without significant changes in position. Two specimens were damaged when separated from the 3D printed cap, thus being excluded from analyses.

#### Volumetric analysis by micro-CT

**Figure 4:**
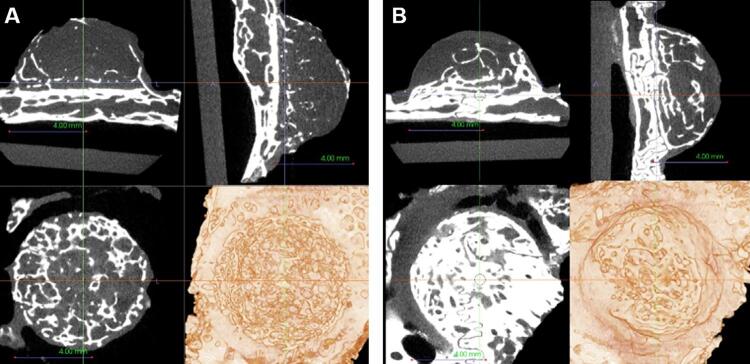
Cross-sectional and 3D rendering of micro-CT images of a specimen at week 6

**Figure 5:**
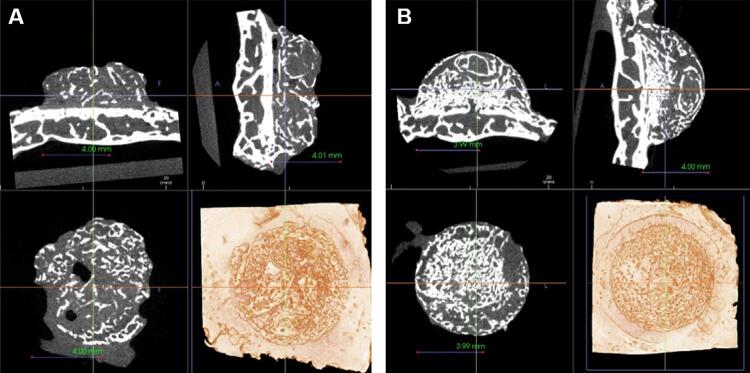
Cross-sectional and 3D rendering of micro-CT images of a specimen at week 12

From micro-CT scans, we observed that most specimens showed a pattern of new bone formation upward from the calvaria, especially those in group I (Figure 4). However, group II (to which BMP was applied) showed a more evenly new bone formation throughout the area beneath the cap (Figure 5). The region of interest (ROI) analysis showed no significant difference between groups at week 6 and week 12 (Table 1). We also verified no difference between groups regarding bone formation over 300 HU at weeks six and twelve, but this variable was significantly higher in group II at week 12 than at week 6. Regarding the upper portion of the cap space, bone formation significantly differed between groups at week 12, with an ICC for volume measurements equal to 0.992 (95% CI: 0.912 – 0.997).

Radiopaque findings, which means new bone formation, are observed from the experimental group I (A) and II (B).

New bone formation is observed more evenly distributed at experimental group I (B) than group II (A).

**Table 1:** Quantitative analysis of the volume of new bone formation

				(10^-3^ml, mean ±SD)	
		Volume of new bone formation at 6 Weeks		Volume of new bone formation at 12 Weeks	

Radiopacity	Area beneath the cap	Group I (n=4)	Group II (n=4)	Group I (n=4)	Group II (n=4)
> 300 HU	Whole	19.5±5.92	23.75±10.87*	27±6.73	53.75±22.50*
	Upper portion	2.25±3.20	4.5±2.52	5±2^†^	18.25±6.99^†^
> 1000 HU	Whole	12±5.94	14.25±7.37	11±4.08	29.5±15.20
	Upper portion	1±1.41	2.75±1.71	2±0.82^§^	8±3.46^§^

*,†,§ indicates statistical significance p<0.05.

#### Histologic analysis

**Figure 6:**
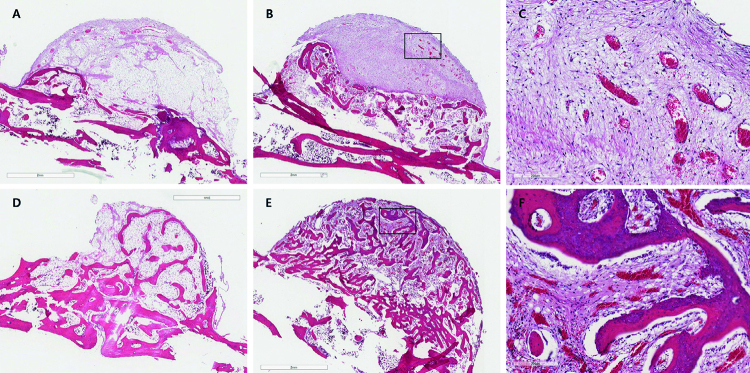
Histological images of the specimen shows new bone formation in the area below the cap, especially at the site of the rhBMP-2 application

Micro-CT analysis indicated the presence of connective tissue following the cap shape and the formation of new bone tissue. Whereas in group I new bone tissue was formed above the calvaria surface, in group II bone was formed within the cap (Figure 6).

Specimen from experimental group I at week 6 (A, magnification x1.5, scale bar 2mm) and 12 (B, magnification x1.5, scale bar 2mm; C, box area in image B, magnification x10, scale bar 200mm), and experimental group II at week 6 (D, magnification x1.5, scale bar 2mm) and 12 (E, magnification x1.5, scale bar 2mm; F, box area in image E, magnification x10, scale bar 200mm).

## Discussion

Ti-6Al-4V titanium alloy is the most widely used metal with commercially pure titanium, providing advantages such as high strength, low density, high fracture toughness, excellent corrosion resistance, and superior biocompatibility.^11^However, when employing such alloy, one must consider the toxicity related to the possible release of vanadium or aluminum ions inherent to changes in its manufacturing process.^12^

Titanium materials are also actively used in 3D printing. A study conducted with a micro-implant produced by DMLS placed in the human body and removed after two months found a 69.51% bone-to-implant contact ratio.^13^Another study comparing a 3D-printed titanium alloy produced by SLM with a control group verified a good suitability for human primary osteoblast-like cells in the printed model through the cell proliferation XTT assay.^14^However, printouts obtained through 3D printing require washing with an organic solvent, so that these substances remnants may adversely affect the surrounding issues.^15^Thus, quality control is as important as the raw material in the entire printing process.

For presenting similar behavior to that of primary calvarial osteoblasts, our cytotoxicity test was conducted with MC3T3-E1 cells.^16^ Cell count was significantly different between groups after 48 hours of culture, indicating that neither raw materials ingredients nor the printing process were harmful. A recent study found evidence on the stability and acceptability of 3D printing using titanium materials, boosting studies investigating its applicability to various clinical fields.^17^

As printed materials seems to present a rougher surface when compared to conventionally-cut materials, metal 3D printing requires biocompatibility evaluation. With this end, we performed a cytotoxicity test, verifying similar biocompatibility values between printed and conventional-cut specimens, thus indicating no problems in adapting to the living body. However, printouts may require an additional process such as polishing. Some studies reported the risk of cracking and long-term fatigue in 3D-printed outputs, calling attention for their use in clinical applications.^18,19^

The volume of new bone formation was measured in the field of interest ( >300 and 1000 UH) using a computer software and based on previous studies.^20,21^We verified a significant increase in new bone formation in group I from six to twelve weeks, differently than group II. The morphometric analysis verified a more even distribution of new bone beneath the cap in group II. Likewise, group II also showed significantly more new bone formation within 2mm beneath the cap in the volumetric analysis; that is, at the part farther away from the host calvarial bone. These findings allow us to infer that BMP has an osteoinductive function.^22,23^

Although the ideal dose of rhBMP-2 should be standardized to maximize the osteoinductive effect of this protein depending on bone defect size and morphology, this is not an easy task.^24,25^A study on critically-sized bone defects (8mm diameter) in rat calvaria clearly verified bone formation upon the application of rhBMP-2 from 2.5-20mg using an absorbable collagen sponge,.^23^Another study investigating the application of 20mg of rhBMP-2 with β-tricalcium phosphate on rabbit mandible defects reported effective bone formation.^26^

Although our study was not aimed to determine the ideal concentration of rhBMP-2 for bone regeneration, we verified significant results in the group receiving the protein at 0.1mg/ml. When applied with an absorbable collagen sponge, rhBMP-2 retention to the application site was shorter (mean residence time from 4-8 days in the rat).^27^

Based on a study that reported the possibility of using telopeptide collagen as a BMP carrier,^28^we applied BMP with a collagen plug to group II, demonstrating better bone regeneration patterns than those observed in group I. Another study investigating the insertion of a resorbable collagen plug to critical-sized defects in rats in comparison to no insertion found better bone regeneration results with the collagen plug.^29^In our study, micro-CT analysis indicated the presence of some bone regeneration even in group I – to which BMP was not applied.

In our study, the group that received rhBMP-2 presented more even bone formation. However, further information on the appropriate concentration of BMP and carrier materials are required.^30^ The cellular activity of bone formation may be better understood by identifying changes in osteoblast activity,^31^ which in turn can be assessed using immunolabeling of biomarkers, such as alkaline phosphatase (ALP) and osteocalcin.^32^

Our study has some limitations. At euthanasia, some caps put into the groove formed in animals’ skull were not stable, which may have negatively affected surgical site stability. Thus, these cases were excluded from the analyses. We reinforce the need for further studies on bone regeneration to ensure the stability of the implant throughout the experiment. Moreover, our study sample consisted of a limited number of animals, besides not being conducted in the human body, requiring more clinical studies on the subject.

## Conclusions

Our results indicate that 3D-printed titanium products presented satisfactory biocompatibility and promoted the formation of new bone when applied with rhBMP-2. These findings suggest that 3D-printed implants may be used to induce bone formation in a desired patient-specific shape.
